# Global Invasion Risk Assessment of *Prosopis juliflora* at Biome Level: Does Soil Matter?

**DOI:** 10.3390/biology10030203

**Published:** 2021-03-09

**Authors:** Mohammed A. Dakhil, Ali El-Keblawy, Mohamed A. El-Sheikh, Marwa Waseem A. Halmy, Taoufik Ksiksi, Walaa A. Hassan

**Affiliations:** 1 Botany and Microbiology Department, Faculty of Science, Helwan University, Cairo 11790, Egypt; mohamed_dakhil@science.helwan.edu.eg; 2 Department of Applied Biology, Faculty of Science, University of Sharjah, Sharjah P.O. Box 27272, United Arab Emirates; 3 Botany & Microbiology Department, College of Science, King Saud University, P.O. Box 2455, Riyadh 11451, Saudi Arabia; melsheikh@ksu.edu.sa; 4 Department of Environmental Sciences, Faculty of Science, Alexandria University, Alexandria 21511, Egypt; maraw.w.halmy@alexu.edu.eg; 5 Biology Department, United Arab Emirates University, Al Ain 15258, United Arab Emirates; tksiksi@uaeu.ac.ae; 6 Botany and Microbiology Department, Faculty of Science, Beni-Suef University, Beni-Suef 62511, Egypt; azmeyw@gmail.com

**Keywords:** invasion risk assessment, temperature variability, habitat suitability, global biomes, MaxEnt, conservation priority

## Abstract

**Simple Summary:**

Invasive plant species are one of the major threats to biodiversity and cause the loss of natural habitats. Invasive Mesquite plant was continuing to spread all over the world and invaded most of the forest-shrubland biomes. We aimed to evaluate the contribution of soil and huaman-influence factors and climatic factors to the distribution dynamics and expansion of Mesquite invasive plant. Also, it aimed at ranking the threatened areas in each global biome. Our findings revealed that the invasion risk increases with temperature, soil alkalinity, and clay fractions. This study would provide great insights into prioritization and management guidelines to monitor the expansion and invasion risk of Mesquite plant in the whole world.

**Abstract:**

*Prosopis juliflora* is one of the most problematic invasive trees in tropical and subtropical regions. Understanding driving forces affecting the potential global distribution would help in managing its current and future spread. The role of climate on the global spatial distribution of *P. juliflora* has been well studied, but little is known about the role of soil and human impacts as potential drivers. Here, we used maximum entropy (MaxEnt) for species distribution modelling to understand the role of climate (C), soil (S) and human impacts (H), C+S, and C+S+H in controlling the potential invasion range of *P. juliflora*, and to project its global potential invasive risk. We defined the top threatened global biomes, as predicted by the best-selected model. The incorporation of the edaphic factors improved the model performance and enhanced the accuracy of the outcome. Our findings revealed that the potential invasion risk increases with increases in mean temperature of the driest quarter (Bio9), soil alkalinity and clay fractions. Arid and semi-arid lands are at the highest risk of invasion than other moist biomes.

## 1. Introduction

The increase in human travel and trade has accidentally or intentionally increased the spread of many species from their native ranges [1]. The increasing numbers of introduced invasive species and their potential to change the social-ecological systems have been considered a major global change component [2,3,4]. The ecological impacts of the introduction of invasive plants include, for example, degradation of ecosystem structure and function, change in community composition, and loss of species diversity [5,6]. Invasive exotic plants have become among the major challenges facing social-ecological systems, especially rangelands and livestock [4]. The social-ecological impacts of introduced invasive plants would be severe without applying effective and preventive management approaches [7].

*Prosopis* L. (Fabaceae) has 44 species of trees and large shrubs [8]. *Prosopis juliflora* is the hardiest and most resilient xerophytic tree of the genus, originated from North America (Mexico) or Central America (Costa Rica, El Salvador, Guatemala, Honduras, Nicaragua, Panama). It is naturalized and invaded several tropical and sub-tropical regions worldwide at an alarming rate [1,9]. In addition, this species is surviving and thriving in the harsh conditions of the hyperarid Arabian deserts, where annual average rainfall is less than 100 mm and average temperatures reach above 40 °C during summer [10,11,12,13,14,15]. The introduction was for different purposes, including for example, combating desertification and land reclamation, greening deserts, and its utilization as sources of animal feed, especially pods that represent a considerable part of goats’ diet, biofuel, timber, shelter, building material and furniture for local farmers [1,8,16]. However, after its introduction, *P. juliflora* turned to be one of the most problematic trees globally, particularly in the rangelands, croplands, and forests, and threatens the pastoral livelihoods and ecosystems. The IUCN has considered *P. juliflora* as one of the worst 100 invasive alien species globally [17,18].

Several environmental factors helped the rapid invasion of *P. juliflora* to new ranges. For example, the hardy nature of *P. juliflora* and its ability to tolerate a wide range of temperature, water/soil quality, and humidity makes it among successful invasive species to the hot, dry, and wet tropical and subtropical regions. Moreover, plants’ ability to adopt a wide range of climatic and soil conditions, high coppicing ability, effective dispersal mechanisms and production of allelochemicals accelerated the invasion rate of *P. juliflora* [19,20]. Furthermore, this species propagates both sexually with vast numbers of viable seeds and vegetatively by adventitious buds present on the shallow roots. The dormant seeds build long-lasting seed banks as a bet-hedging strategy against unfavorable years of below-average precipitation or drought [21]. Such diversity in the propagation methods enabled *P. juliflora* to successfully colonize new areas [22,23].

Among several algorithms, maximum entropy (MaxEnt) was regarded as one of the best bioclimatic species distribution models (SDMs). MaxEnt uses presence-only data to predict species distribution [24]. Several studies have used SDMs and MaxEnt to predict the invasion of introduced plants at a continental scale, based on their occurrence records and preferable environmental variables. Most studies have used climatic, environmental variables to model invasive plant distribution [25]. However, there are numerous environmental variables that can affect the ability of models to predict the distribution of invasive plants [26]. For example, the use of other environmental factors, such as soil, water and human impacts, in addition to the climatic data, should provide higher predictive powers in SDMs [27].

Several researchers have used the SDM to study the impacts of climate change on future invasion and distribution range of *P. juliflora* at local and regional scales [7,28,29]. For example, Wakie et al. [28] concluded that Moderate Resolution Imaging Spectroradiometer (MODIS) vegetation indices and species occurrence points with MaxEnt modeling software could be used to quantify the current distribution of *P. juliflora*. Moreover, [29] studied the effect of global warming on the distribution of *P. juliflora* in its introduced range using SDMs and MaxEnt approach. They concluded that more than 87% of the model’s variations were explained in the light of the annual mean temperature, annual precipitation, and temperature means diurnal range. Besides, it has been reported the intolerance to low temperatures was the most critical factor that limits the global distribution of *P. juliflora*; the level, duration, and frequency of frosts limit the reproduction and growth of this species [9].

Several studies have reported the importance of using combinations of predictor variables to understand the complex interplay between biological invasions and global environmental distribution to understand plant invasion [30]. For example, several models indicated that soil factors could affect the physiological performance of invasive plants and, therefore, could affect their distribution range [31,32]. Despite the fact that several studies have studied the effect of climatic factors on the potential distribution of *P. juliflora*, few have assessed the roles of soil attributes as well human pressures as potential environmental drivers that could affect the distribution of this species. For example, Nascimento et al. [33] have recently shown that both soil properties and human activities could be potential drivers for the distribution, proliferation, and invasion ability of *P. juliflora*. They concluded that some life-history traits of this species could help it to benefit from human activities. For example, grazing animals could increase seed dispersal, germination, and enhance soil fertility, enhancing the dispersal of *P. juliflora* to new habitats, even in less-fertile degraded lands. In addition, [28] reported that the inclusion of soil and related hydrologic parameters in the spatial distribution models could provide more insights into the current and potential distribution of *P. juliflora*.

Furthermore, Abbas et al. [34] modeled the spatial distribution of *P. juliflora* along an environmental gradient in Upper Egypt. They concluded that elevation and distance from the road, as an indicator of human disturbance, significantly correlated with its ability to spread in new habitats. However, little knowledge is known about human impacts and soil variability on the potential distribution of *P. juliflora* at a global scale [32]. We expect that incorporating soil properties and human impact data into SDMs would provide more accurate and precise predictions of the potential distribution, hence the invasion risk of *P. juliflora*. Therefore, the objectives of the current study were to estimate the global potential invasion risk of *P. juliflora* using three different models: climate (C), climate and soil (C+S), and climate, soil, and human influence (C+S+H). The study also aimed at ranking the top threatened global biomes based on the potential invasion suitability produced by the best-selected model.

The combination of more predictor variables would help explain the complex interplay between biological invasions and the global environment and the socio-economic processes and provide more insights towards effective management of the invasive *P. juliflora*. Moreover, incorporating soil quantity data into SDMs would provide more accurate and precise predictions of the invasion risk (i.e., the potential distribution of *P. juliflora* under current conditions of climate and soil. To the best of our knowledge, this is the first study involving potential evapotranspiration and aridity index, wind speed, solar radiation variables along with soil variables simultaneously into the SDM to investigate the global pattern of distribution of *P. juliflora*. These variables can potentially affect the growth and potential distribution of plant species, and this incorporation could improve the prediction of plant distribution [35]. The overall result would help determine the best predictor variable(s) that could explain the potential distribution of *P. juliflora*.

## 2. Materials and Methods

### 2.1. Global Distribution Data

**Figure 1:**
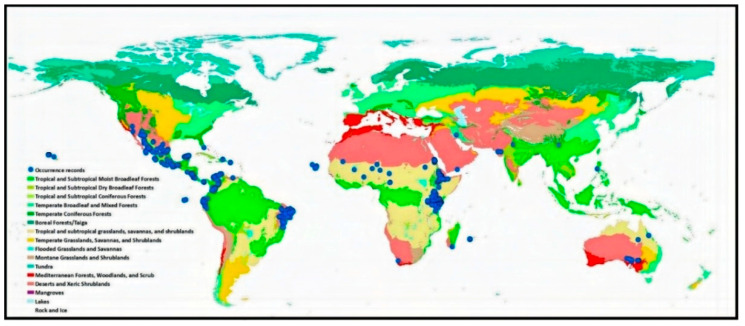
Global distribution of the sixteen terrestrial biomes and the occurrence records of *P. juliflora*.

We obtained the occurrence data of P. juliflora from the Global Biodiversity Information Facility (GBIF.org, https://doi.org/10.15468/dl.sgpgg0 (accessed on 13 December 2018)). The downloaded database hosts 1752 geo-referenced records (1950–2018), including coordinates. The source of these occurrence data was human observations and preserved specimens. We verified the records using ArcGIS 10.3 [36] to remove records outside the shapefile [36] of the world map or located in water and deleted duplicate geographical records. This resulted in 1173 distribution points representing 35 world countries (Figure 1 and Supplementary A, Table S1), which were reduced further into 866 records representing 34 countries after deleting the reciprocated missing values of the resampled environmental variables of climate, topography, and soil.

### 2.2. Environmental and Human Variable Predictors

#### 2.2.1. Bioclimatic Variables

To predict the potentially suitable habitats for *P. juliflora* in the world, we downloaded the nineteen standards bioclimatic variables of the current climate (1970–2000) from the WorldClim 2.0 along with wind speed and solar radiation (http://www.worldclim.org; accessed on 7 April 2019 [37]) at a spatial resolution of 2.5 arc-minutes (~5 km × 5 km at the equator). We generated the mean raster layers of the monthly layers of wind speed and solar radiation using the spatial analyst toolbox in ArcGIS 10.3 [36].

The data of potential evapotranspiration (PET), actual evapotranspiration (AET) and aridity index (AI) were downloaded from CGIAR-CSI Global database ([38]; www.cgiar-csi.org; accessed on 16 May 2017) at a spatial resolution of 30 arcseconds (~1 km at the equator), and then resampled into resolution 2.5 arc-minutes using ArcGIS10.3 [36].

#### 2.2.2. Edaphic Variables

Nine quantitative variables representing the soil physical and chemical properties were downloaded from the ISRIC-World Soil Information database (ftp://ftp.soilgrids.org/data/aggregated; accessed on 27 March 2019 [39] at a depth of 0–2 m and a spatial resolution of 30 arcseconds. The mean raster layers of the different soil depths were generated using the spatial analyst toolbox and then resampled into a resolution of 2.5 arc-minutes using ArcGIS10.3 [36].

#### 2.2.3. Human-Activity Variable

The Global Human Influence Index Dataset of the Last of the Wild Project is a global dataset of 1-km^2^ grid cells [39] was created from nine global data layers covering human population pressure, human land use, and infrastructure (land use/land cover, built-up areas and nighttime lights), and human access (roads, railroads, coastlines, and navigable rivers). The human influence data was resampled into a resolution of 2.5 arc-minutes using ArcGIS10.3 [36].

### 2.3. Multicollinearity, Model Construction, and Predictions

All the environmental data (34 variables; see Supplementary A, Table S2) were extracted from the raster layers using species occurrence records in ArcGIS 10.3 [36] for multicollinearity analysis of the environmental predictors to avoid overfitting of models, poor model performance, and misleading interpretations. The climatic and edaphic predictors were analyzed separately, and only the predictors with correlation coefficient “Spearman” or “Pearson” |r| ≤ 0.7 (Supplementary A, Table S3 and Table S4) and eco-physiologically meaningful were selected for the model development [40]. We used IBM SPSS v.21.0 [40] to perform multicollinearity analysis.

We converted 15, out of the 34 environmental predictors (Table 1), in addition to human influence index predictor layers, into ASCII format before their use for MaxEnt models. We used the resolution of 2.5 arc-minutes to allow more flexibility of the interactive geographical relationship between the species and its environment; this resolution was used previously in similar studies concerned with global distribution modelling of invasive plant species such as *Phragmites australis* [41] and *Parthenium hysterophorus* [42].

We used MaxEnt version 3.4.1k [43] to map the potential distribution of *P. juliflora*. The default settings were used, i.e., the maximum number of iterations (500), the replicated run type (cross-validation), and the output of the logistic. The accuracy of the models was tested by partitioning the data into 75% training and 25% testing subsets. Furthermore, the regularization setting was optimized to 2.5 to improve models’ transferability across space and reduce the likelihood of models overfitting [44,45,46,47].

We built three different models: (1) model C (based on climate only), (2) model C+S (based on both climate and soil variables), (3) model C+S+H (based on climate, soil, and human influence variables). Then, we ran MaxEnt using occurrence records with each of the three models separately.

To evaluate the model accuracy, we calculated the True Skill Statistic (TSS), which is threshold dependent. Therefore, we used two settings in MaxEnt: write background predictions and the minimum training presence threshold (MTP). The latter is recommended to study invasive species distribution and risk assessment, providing a greater area of invasion suitability [45,48].

The logistic output of habitat suitability provides the probability of *P. juliflora*, which ranges from 0 to 1. MaxEnt may give high-prediction values for environmental conditions outside the range of the target species. Hence, to avoid overestimation, we used only those pixels with values ≥ 0.5 of the continuous suitability index, representing high invasion suitability [9,43,49]. The invasion suitability values were also extracted using the number of grid cells with suitability ≥ 0.5 in each global terrestrial biome [50] (Figure 1 and Supplementary A, Table S5) using spatial join in ArcGIS 10.3 [36]. Then, we ranked the world terrestrial biomes based on the number of grid cells with a suitability score ≥ 0.5 to determine the invasion risk level.

### 2.4. Model Performance and Evaluation

The area under the curve (AUC) values of the receiver operating characteristic (ROC), helped us to compare the performance of the three models. The AUC is usually commonly used in evaluating multiple MaxEnt models. An AUC value closer to 1 indicates better model performance [42,51]. Sensitivity and True Skill Statistic (TSS) are also appropriate measures of the model accuracy to evaluate the model performance [52,53]. Unlike the AUC, sensitivity and TSS are threshold-dependent, and the latter accounts for both sensitivity and specificity, with values ranging from −1 to +1. TSS is the sensitivity (percent of presences correctly predicted) + specificity (percent of absences correctly predicted)—1 of model predictions [52]. It is more likely to make a correct prediction when the species is present (means has higher sensitivity) [54].

Jack-knife tests and permutation importance provide an approach to evaluate the efficiency or predictive power and the relative importance of predictors where each variable is excluded in turn, and the model of each predictor in isolation is compared with a model comprising the remaining predictors [51]. The importance of the variables in the invasion suitability models was calculated by randomly permuting training presence and background data, i.e., permutation importance, which is an essential indicator for the strong model dependence on a particular variable [44,55]. The response curves show how environmental variables affect the prediction of invasion suitability. The curves show how the predicted probability of presence (invasion suitability) changes as each environmental variable is changed, keeping all the other environmental variables at their average values [56].

## 3. Results

### 3.1. Model Performance

All three models showed good fits and high performance in the prediction of the species distribution. All values of the sensitivity and AUC of the models were > 0.9; meanwhile, the TSS values of the three models were larger than 0.5; meaning a good performance (Table 2). Furthermore, the AUC values of the models “C+S” and “C+S+H” were slightly larger than those of “C” (Table 2). We represented the output of the climate and soil model (C+S) due to the higher relevant contribution and relative importance of the soil variables (Table 2) in the prediction of the species distribution.

### 3.2. Global Distribution and Potential Invasion Suitability of P. juliflora

**Figure 2:**
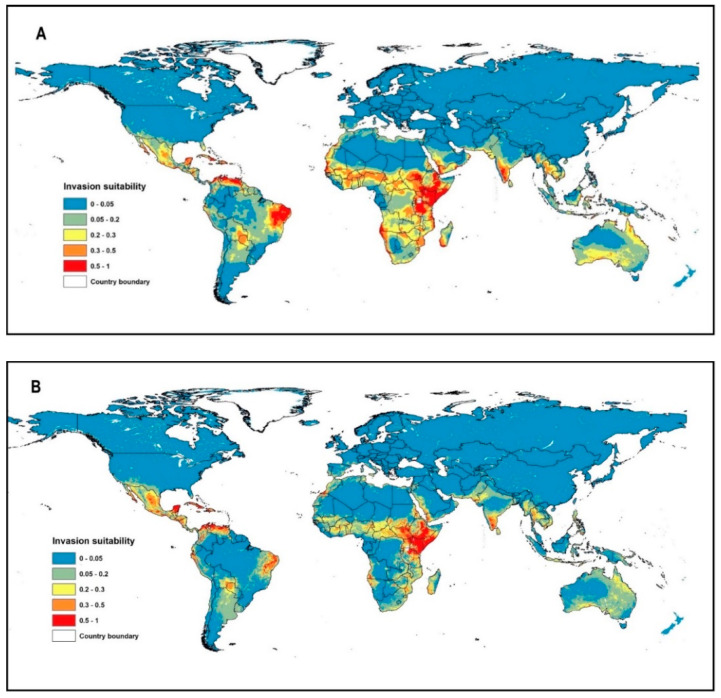

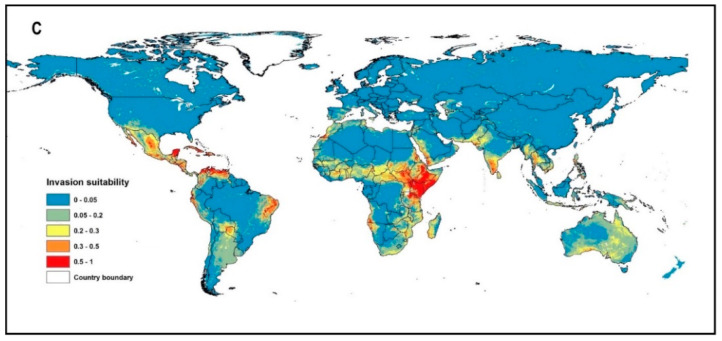
Global invasion risk (potential habitat suitability) of *P. juliflora* according to the three different models: (**A**) Climate model, (**B**) Climate and soil model (**C**) Climate, soil, and human influence mode.

Most of the occurrence records (99%) were located in the arid and hyper-arid regions (Aridity index < 0.2, Supplementary A, Table S2), which are located mainly in the tropical and subtropical biomes (Figure 1). The invasion of suitable areas was reduced when soil or human influence variables were incorporated into the model (Figure 2A–C). Moreover, the human influence factor was contributed only by 0.1% to the potential distribution, indicating that human influence is not an important variable to the potential invasion suitability.

### 3.3. P. juliflora Invasion Suitability and Environmental Variables

Based on the percent contribution of the predictor variables that were generated by MaxEnt model C+S, the most important factors defining the potential global distribution of *P. juliflora* were temperature seasonality (Bio4, 43.1%), PET (10.7%), soil pH (25.6%), and soil clay texture fraction (9.3%) (Table 2). The responses of the remaining variables that showed less importance were shown in Supplementary B, Figure S2. Regarding the permutation importance, the maximum influence on the potential invasion suitability was for temperature seasonality (Bio4, probability of the presence was 42%), followed by the temperature of the driest quarter (Bio9, 21.1%), then soil pH (17.5%), and finally by soil clay texture fraction (5.9%).

**Figure 3:**
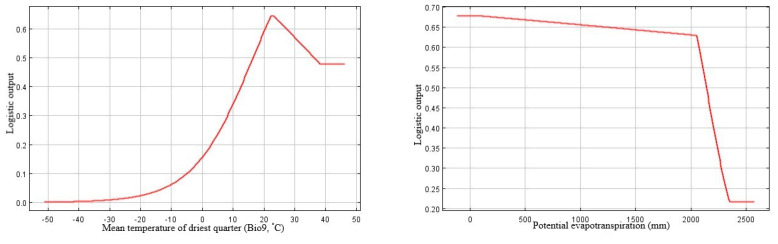

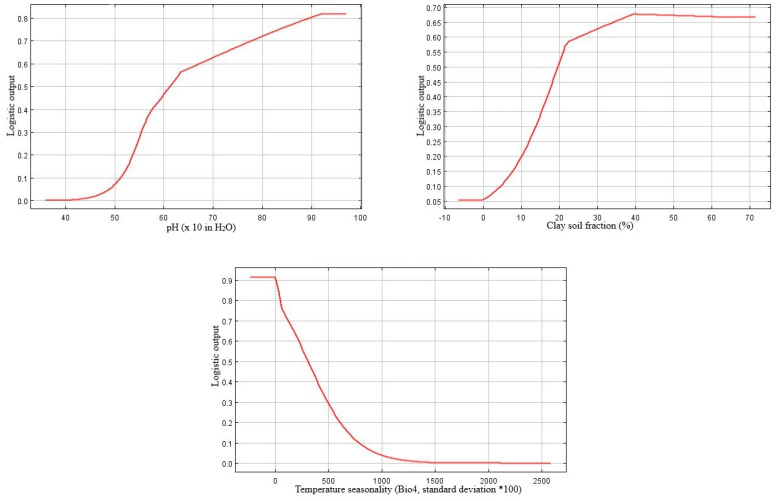
Response curves of the most influential climatic and edaphic predictors generated by MaxEnt (Climate + Soil model). Bio4: temperature seasonality (standard deviation *100); Bio9: Mean temperature of the driest quarter (°C); PET: potential evapotranspiration; PHIHOX: soil pH × 10 in H_2_O; CEC: CLYPPT: soil texture fraction clay.

The probability of the presence of *P. juliflora* decreased in response to temperature seasonality (Bio4), gradually up to a variation of 13% (Figure 3). The likelihood of *P. juliflora* presence increased gradually with the increase of the driest quarter temperature (Bio9) at a suitable range (approximately 21–23 °C); the optimal value is approximately 22 °C. Above 23 °C, the invasion suitability of *P. juliflora* showed a gradual decline (Figure 3).

Regarding the climate-based model (Model C), the invasion suitability decreases gradually with the increase of aridity index up to an approximate value of 0.42 (semi-arid conditions) above which the suitability is very low and becomes constant (Supplementary B, Figure S2). With the increase of solar radiation, the invasion suitability increases up to an approximated maximum value of 19,500 kJ m^−2^ day^−1^, above which a sharp decline of the logistic suitability occurs (Supplementary B, Figure S2). The overall response of invasion suitability prediction for the wind speed is positive above 18 m s^−1^; the logistic invasion suitability increases with the increase of wind speed up to the maximum approximated speed of 120 m s^−1^ (Supplementary B, Figure S2).

Regarding the soil suitability for the occurrence of *P. juliflora*, the model “C+S” results showed that soil pH and soil clay fraction were the key soil factors determining the distribution of this species along with the climatic factors mentioned above. The potential invasion suitability increases with soil pH; the most excellent presence probability was at an approximate pH of 9.2 (Figure 3). This result suggests that *P. juliflora* prefers alkaline habitats. There is a sharp increase in the presence probability of *P. juliflora* with the increase in clay content up to 22%, after which there is a gentle linear increase of the occurrence till 70% (Figure 3). Moreover, there is a linear increase in the occurrence suitability of *P. juliflora* with the increase in the available soil water capacity from 2% up to 11.5%; above that; there is no change (Supplementary B, Figure S2).

### 3.4. Potential Invasion Risk at Biom Level

**Figure 4:**
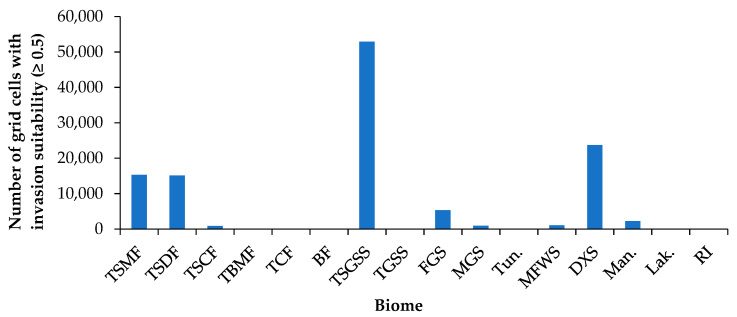
Invasion risk to global biomes with high habitat suitability (≥0.5) for the invasion of *P. juliflora*. Abbreviations: TSMF = Tropical and Subtropical Moist Broadleaf Forests; TSDF = Tropical and Subtropical Dry Broadleaf Forests; TSCF = Tropical and Subtropical Coniferous Forests; TBMF = Temperate Broadleaf and Mixed Forests; TCF = Temperate Conifer Forests; BF = Boreal Forests/Taiga; TSGSS = Tropical and subtropical grasslands, savannahs, and shrublands; TGSS = Temperate Grasslands, Savannahs, and Shrublands; FGS = Flooded Grasslands and Savannahs; MGS = Montane Grasslands and Shrublands; Tun. = Tundra; MFWS = Mediterranean Forests, Woodlands, and Scrub; DXS = Deserts and Xeric Shrublands; Man. = Mangroves; Lak. = Lakes; RI = Rock and Ice.

The invasion suitability of *P. juliflora* varied remarkably among the main biome types (Figure 4). The highest invasion suitability is in the tropical and subtropical grasslands, savannahs, and shrublands (TSGSS, 52,896 grid cells), followed by the deserts and xeric shrublands (DXS, 23,713) (Figure 4, and Supplementary A, Table S5). The tropical and subtropical moist and dry broadleaf forests (TSMF and TSDF) have moderate chances of being among high invasion risk biomes. Furthermore, there is a lower chance of flooded grasslands and savannahs (FGS) and mangroves among invaded high-risk biomes (Figure 4). The biomes that have the lowest chance for invasion are Mediterranean forests, woodlands, and scrub (MFWS), montane grasslands and shrublands (MGS), tropical and subtropical coniferous forests (TSCF), and lakes. It is worth noting that the global biomes showing high potential invasion suitability fell in the order: TSGSS > DXS > TSMF > TSDF > FGS > mangroves > MFWS > MGS > TSCF > lakes (Figure 4).

## 4. Discussion

### 4.1. Potential Distribution of P. juliflora and Invasion Suitability Models

Climate is a key factor in determining the species capabilities to conquer and invade new areas [7,29]. However, other non-climatic factors are as important as the climate in influencing species abilities to invade new areas, including physical and chemical properties of soils, moisture availability, topographic features, and human-induced disturbances [35,57]. The earlier studies that assessed the invasive abilities of *P. juliflora* to new range depended mainly on climate [1,7]. For example, EPPO [9] had used MaxEnt in an ensemble modelling with other models to explain the potential distribution of *P. juliflora*. MaxEnt model showed high accuracy with similar contribution and response of the climatic moisture index (18%) [9], and this agrees to our results of the contribution and response of the evapotranspiration (15%). On the other hand, the model also showed high contribution of the temperature of the coldest month (64%), but this differ from our results which revealed that temperature seasonality (49.6%). This difference may be attributed to using different predictors, but our study showed more precise predictions of the invasion suitability because we built our best model on climate and soil. Furthermore, we selected the relevant variables based on the occurrence data of the species where it was distributed in the arid and hyper-arid regions, so temperature of the driest quarter and temperature seasonality are more relevant bioclimatic variables than other variables. However, incorporating the edaphic factors in our models showed a higher performance and produced more accurate predictions. The suitable areas for invasion with *P. juliflora* were reduced when soil or human influence variables were incorporated into the model. Provided that suitable climatic conditions are available, edaphic factors can set the ecological boundaries that restrict species distributions and determine community composition [58]. Adding the soil variables, particularly soil pH and clay fraction, showed high relevant contribution and relative importance in the prediction of *P. juliflora* distribution. For example, the increase in soil pH (i.e., alkalinity) markedly increased the probability of the presence of *P. juliflora*. However, the probability decreased above pH 9.0. It has been reported that the suitability of soil substrates for germination and seedling growth of *P. juliflora* was significantly reduced above pH of 9.0 [58]. Soil texture, pH, and moisture content are important ecological conditions that significantly influenced native and invasive species distribution in the arid and semi-arid ecosystem [59]. Other biological factors that can play a significant role locally in the invasion potential, which was not accounted for at the global level, are livestock and birds in long-distance seed-dispersal [60,61].

In general, non-climatic factors related to human-induced activities such as land-use changes, the establishment of road networks, transportation, etc. facilitate introducing species to new areas and increase invasion susceptibility [62]. In our study, however, the human influence indicator did not contribute significantly to the models’ performance predicting the distribution of *P. juliflora* at the global scales. The significance of the human activities and the associated disturbances in influencing the potential invasion suitability of *P. juliflora* could be more significant at a local rather than a global scale. For example, Abbas et al. [34] found that the probability of *P. juliflora* was higher in areas closer to roads. The Human Influence Index (HII) used in the current study as an indicator of human disturbances on the natural ecosystems was derived based on measures urbanization level measures, accessibility by transportation means, degree of landscape modification, and access to electricity and power networks. The elements of the HII are higher in the developed countries that are mostly located outside the range of occurrence of *P. juliflora*, whereas areas that are suitable climatically to the occurrence of *P. juliflora* are located in the arid and hyper-arid developing countries (99% of the occurrence records); mainly in the southern hemisphere. Therefore, the index did not contribute much in connecting human-induced disturbances to the invasion potential of *P. juliflora* at the global scale.

### 4.2. Environmental Drivers Best Explain the Invasion Suitability of P. juliflora

Our results of the response curves revealed that the increase in temperature of the driest season would increase the invasion risk or expansion of *P. juliflora*. The projected global increase by about 1–3 °C in temperature by the end of this century would trigger shifts in arid/humid climate zones worldwide [63]. The projected conversion of some areas that are currently considered humid and semi-arid into arid zones due to global warming will provide more suitable areas for the invasion of *P. juliflora* [64,65]. In addition, climate scenarios projected an increase in the temperatures of the driest seasons and in the aridity of many parts of the humid and semi-arid regions, which again could create more suitable areas for the invasion of *P. juliflora* under climate change.

Our study’s outcomes suggested that the optimum temperature for the logistic potential distribution or presence suitability was 22 °C. Earlier studies have revealed the ability of *P. juliflora* to withstand temperatures up to 50 °C [10,14,15]. Moreover, the increase in temperature was associated with increases in the germination rate [8,66]. The accumulation of a high density of seeds (ca. 60 million seeds ha^−1^ yr^−1^) in the soils can enable *P. juliflora* to regenerate at higher temperatures successfully and under light conditions [66,67,68]. It is worth noting that temperature interacted with light to regulate seed germination of *P. juliflora*, provided that moisture is available. This indicates that disturbance could bring seeds to the upper layers, where both light and temperature conditions are suitable for germination [69]. Livestock and flooding are two crucial dispersal agents of seeds; both enhance seed germination and seedling establishment and facilitate the expansion and invasion of *P. juliflora* [70]. According to the plant-soil feedbacks hypothesis, both leguminous plant and microbes affect each other to improve soils’ physical and chemical properties [71]. *P. juliflora* can improve soil properties through soil microorganisms, living free in soils or forming a symbiotic relationship in the plants’ nodules. Besides, soil microflora can increase litter decomposition that increases clay fraction and water holding capacity. According to our models, the increase in soil moisture and clay fraction can enhance the invasive ability of *P. juliflora.* The amelioration of soil characters by *P. juliflora* is particularly important in sandy soils in arid biomes, which are characterized by lower fertility and higher pH [10,14]. Soil fertility, including N, was significantly greater beneath and around *P. juliflora* canopies than in soils away from them [14].

### 4.3. Potential Invasion Risk

#### 4.3.1. At the National Level

Ninety percent of the total world countries where *P. juliflora* currently occurs showed high suitability of invasion risk. This finding is supported by earlier studies [1,9].Surveys and monitoring of the population dynamic of this invasive species are needed, especially in countries with no current records. Moreover, there should be preventive approaches for the arrival and propagation of this invasive species, especially in countries with no current records and those predicted to achieve high invasion under climate change [29].

Livestock and wildlife animals help in seed dispersal and germination of *P. juliflora* by increasing chemical and physical scarifications for seeds that have deep physical dormancy [69,72]. For example, in arid and semi-arid north Mediterranean African regions, camels and sheep are common domestic animals that can potentially disperse the seeds of *P. juliflora* and can help in the invasion expansion along animals’ routes [72,73]. In Egypt, these animals disperse *P. juliflora* along Gebel Elba National Park, the southeast part of the Eastern Desert on the border between Egypt and Sudan [72]. Our study defined this region among the threatened regions by the invasion of *P. juliflora* (Figure 2B). Therefore, animal grazing could help expand *P. juliflora* invasion in the southern part of Egypt. Currently, the middle and northern parts of Egypt are not infested with *P. juliflora*. However, these regions and most of the northern regions of the Mediterranean countries could get considerably warmer by 2050 under the moderate and high climatic emission scenarios (RCP 4.5 and RCP 8.5) [29]. Accordingly, both climate change and seed dispersal from south to north, mainly by livestock, could result in the invasion of northern regions of the African counties with *P. juliflora*. It is recommended to monitor and remove any small young populations that appear along animals’ routes in the northern region. Similarly, *P. juliflora* was not considered invasive in Spain until 2014 [1]. This was recently observed in the frost-free coastal and low-lying inland areas of Spain [9], which strongly supports our prediction that Spain is at high invasion risk.

African countries are at high risk of invasion, e.g., Kenya, Ethiopia, Somalia, South Sudan, and Sudan, which introduced this species for economic purposes, mainly for firewood, timber, fodder, and other uses [26,35]. The species provide over 70% of firewood, mainly in rural India and parts of its urban areas [1,74]. The trade-in *P. juliflora* products were valued as above 1.5 million USD in some villages in Kenya [75]. However, after the introduction, *P. juliflora* became invasive and threaten ecosystem services and human health [11]. With the expected climate change and increasing human activities, *P. juliflora* might become more invasive. Therefore, the management and control of *P. juliflora* invasion may pose challenges in some of the affected countries due to some socio-economic factors that facilitate the expansion of *P. juliflora*. Among the socio-economic factors are the economic dependence of local communities in certain nations, especially in developing countries, on the species for production and marketing of charcoal and firewood [1].

In Africa, one of the most critical vital barriers for the effective management of *P. juliflora* species is the lack of strategic planning and prioritization [76]. Adaptive measures to control the invasion of *P. juliflora* vary among the nations and need to consider the ecological settings plus the socio-economic needs. There are huge costs associated with the invasion of *P. juliflora*, such as a reduction in ecosystem services and species diversity. However, this plant provides numerous goods and services, including, for example, the production of honey, edible exudates, gums, fibers, tannins, bio-pesticides, medicinal compounds, biochar, and several forms of biofuels [77,78]. This argued decision-makers to adopt a new cost-effective management strategy depends on the control of *P. juliflora* through sustainable utilization for the control [79]. However, efficient management of the species invasion in countries predicted as edges of high potential invasion may require cross-boundary endeavors for controlling its extension to new regions, mainly when livestock disperse seeds across the borders. Therefore, the findings of the current study would help in strategic planning and prioritization of the invaded land areas based on the potential invasion suitability (i.e., invasion risk level). The accurate maps of invasion suitability for each country provide a promising tool to different stakeholders for the strategic planning towards effective management of this invasive species.

It has been reported that *P. juliflora* is more aggressive in its introduced range as compared to its native range [80]. In its native range, *P. juliflora* coexists with large numbers of other native species; its canopies have strong facilitative effects on neighbor plants [80]. However, *P. juliflora* has interfering effects on the associated plants in its non-native range. For example, this species completely suppressed the native flora in the arid deserts of the UAE, despite it significantly improved the physical and chemical properties of the soil beneath it [14,15]. However, according to our results, the presence probability of *P. juliflora* is higher in some non-native ranges (e.g., Kenya, Ethiopia) than in all countries within the native range. It seems that *P. juliflora* acquired adaptive ecological features enable it to invade the non-native range and determinately affect its ecosystem services and species diversity [9,80]. The detrimental effects of *P. juliflora* in the non-native range further emphasize the importance of adopting an efficient management strategy for controlling this plant through sustainable utilization [79].

#### 4.3.2. At the Biome Level

The global biomes showed remarkable variation in the suitability of the invasion by *P. juliflora*. The tropical and subtropical biomes exhibited the highest suitability compared to other biomes. This can be attributed to the native range of *P. juliflora*, which is the subtropical regions of Central America, northern South America, and the Caribbean [8,80,81]. The tropical and subtropical grasslands, savannahs, and shrublands exhibited the highest suitability, followed by the Deserts and Xeric Shrublands. Other studies e.g., Heshmati et al. [29], concluded that the species pose a higher risk for expansion in these biomes, particularly the Mediterranean region, west and central Asia, North African, and North America. The variation in the invasion suitability of *P. juliflora* in the different global biome types, is due to the broad ecological amplitude, which makes this specie a successful invader in different biomes [19,20].

Our results indicate a high-risk chance for the invasion of *P. juliflora* in the Mediterranean region, especially North African countries and South Europe, such as Spain (Figure 2B). The most critical factor limiting the potential geographical distribution of *P. juliflora* is the minimum temperatures during winter [9]. The level, duration, and frequency of frosts limit the reproduction and growth of this species [1,9]. Severe frost can cause stem and mortality of *P. juliflora* in countries with cold weather [8]. Therefore, there is a doubt about *P. juliflora*’ occurrence in countries of Mediterranean climates, such as Morocco, Algeria, Tunisia, Libya, and Egypt [8,9]. *P. juliflora* was introduced to Spain for vegetation trials in a single location and showed naturalization, but no invasion signs [1]. However, future global warming in the Mediterranean region is expected to exceed global rates by 25% [82]. Besides, temperature increase has been projected to range between 2 °C and 4 °C by the 2080s in Southern Europe with a chance of short or no frost seasons in the Balkans [83]. Such climatic change would increase the chance of the invasive risk of *P. juliflora* to the Mediterranean region.

Also, *P. juliflora* has several adaptive features that enable it to grow well and even flourish in very poor dry hot desert habitats that are commonly considered to be unsuitable for the dominance of many other plant species [11,12,13,74] For example, the deep taproot of this species allows it to secure its water requirements during dry seasons and hence to invade the dry arid lands [74]. In addition, *P. juliflora* was able to tolerate high sun intensity and temperatures of the hyper-arid environment of the Gulf region by avoiding permanent damage of the photosynthetic apparatus that happened by lowering PSII efficiency and dissipating extra light energy through the increase of non-photochemical quenching [12]. Furthermore, the intrinsic water-use efficiency of *P. juliflora* was significantly greater than in the congeneric native *P. cineraria* in the arid deserts [13]. According to our models, higher temperatures, soil alkalinity, and a higher ratio of fine soil particles accelerate the invasion of *P. juliflora*. In the Arab Gulf deserts, the high temperatures coupled with soil alkalinity of the calcareous soils (i.e., higher pH) enhanced the invasion ability of *P. juliflora* invading the region at an alarming rate [10,15,84]. Notably, the growth of *P. juliflora* trees adds more organic contents, which increases the proportion of fine soil particles [10,14]. Such a change in soil texture would exacerbate the invasion rate of *P. juliflora* in such arid deserts. Moreover, *P. juliflora* gains more advantages over its native competitors, making it spread faster in the infested countries [12,13]. The investigations conducted in the Sultanate of Oman, one of the top threatened countries in the desert biome according to our models, indicated that *P. juliflora* germinated faster and greater, even under stress conditions, such as heat and drought, as compared with its native congener *P. cineraria* [84]. In addition, *P. juliflora* produces allelochemicals that suppress seed germination and growth, and even survival, of other species growing in its vicinity [10,15,22,66,85].

The results predicted the dry shrublands and grasslands biomes to be more suitable for *P. juliflora* than moist forest biomes. *Prosopis juliflora* has a more competitive advantage over other native woody plants in arid and semi-arid lands due to its ability to proficiently access and abstract underground water via its efficient root system [74]. Moreover, adult plants of *P. juliflora* have greater resistance to fire than other woody species [86]. Furthermore, the ability of *P. juliflora* to replace native species of the same lifeform and niche [87] is higher in arid regions compared to other moist ecosystems.

Climate change is the key driver in shaping *P. juliflora* distribution patterns continentally and may lead to a shift in biomes boundaries [88]. Moreover, climate change may increase the risk of the proliferation of invasive species [7]. Some biomes are believed to be of relatively higher susceptibility to climate change consequences, especially the tropical rainforest, the deciduous forest, steppe, and grasslands of Asia and North and South America [89]. The ecological sensitivity of these regions will exacerbate the risk of invasion by *P. juliflora*. However, local conditions and factors, particularly soil properties, the substrate’s nature, the existence of natural enemies, and biotic interactions, will define the limits for the species invasion [7].

## 5. Conclusions

Species distribution models that incorporate edaphic factors and climate to project the potential distribution, invasion risk or expansion of *P. juliflora* provide a more precise estimate of the potential susceptible areas to the species invasion. The potential invasion risk of *P. juliflora* increases with the increase of temperature of dry seasons along with soil alkalinity and clay fractions. This confirms that hot arid and semi-arid lands are currently at the highest risk of invasion or expansion than other moist biomes. The top threatened countries that exhibited high invasion suitability are mostly developing countries, most of which are located in Africa. The introduction of the species in some of these countries was for economic purposes, which may pose challenges for control actions. Cross-boundary endeavors for controlling *P. juliflora* expansion to new regions, particularly in countries predicted as edges of high potential invasion, are required to efficiently manage the species invasion, particularly in the tropical and subtropical broadleaf-forests and shrublands. Finally, logical directions of future research on the ranking of world countries with invasion risk based on the area and number of the country’s protected areas with high invasion risk. This would provide great insights into conservation planning.

## Figures and Tables

**Table 1:** List of the predictor variables used in the distribution modelling of *P. juliflora*. All data were standardized to 2.5 arc-min spatial resolution.

Variable	Code	Description	Original Resolution	Source
(1) Climate	Bio_4	Temperature seasonality [Coefficient of Variation (C of V)]	2.5 arc-min	www.worldclim.org
	PET	potential evapotranspiration (mm)	30 arc-sec	CGIAR-CSI Global database
	Solar_rad	solar radiation (kJ m^−2^ day^−1^)	2.5 arc-min	www.worldclim.org
	AI	aridity index	30 arc-sec	CGIAR-CSI Global database
	Wind_spd	wind speed (m s^−1^)	2.5 arc-min	www.worldclim.org
	Bio_8	mean temperatures of the wettest quarter * (°C)	2.5 arc-min	www.worldclim.org
	Bio_9	Mean temperature of the driest quarter (°C)	2.5 arc-min	www.worldclim.org
	Bio_15	precipitation seasonality (C of V)	2.5 arc-min	www.worldclim.org
	Bio_16	precipitation of wettest quarter (mm)	2.5 arc-min	www.worldclim.org
(2) Soil	PHIHOX	soil pH × 10 in H2O	30 arc-sec	ISRIC-World Soil Database
	CEC	cation exchange capacity in cmolc/kg	30 arc-sec	ISRIC-World Soil Database
	CLYPPT	soil texture fraction clay in percent	30 arc-sec	ISRIC-World Soil Database
	ORCDRC	soil organic carbon content in g per kg	30 arc-sec	ISRIC-World Soil Database
	CRFVOL	coarse fragments volumetric in percent	30 arc-sec	ISRIC-World Soil Database
	AWCh	Available soil water capacity	30 arc-sec	ISRIC-World Soil Database
(3) Human Influence	HII	human influence index	30 arc-sec	NASA Socioeconomic Data and Applications Center (SEDAC)

* The term quarter means the mean temperatures during the wettest three months of the year.

**Table 2:** The relative contributions and importance of the climatic, edaphic, and human factors to the potential invasion risk of *P. juliflora*. The sensitivity, true skill statistic (TSS), and the area under the receiver-operating characteristic curve (AUC) indicate the performance or accuracy of the three models generated by MaxEnt. The most important variables and their values are shown in bold.

Variable *	Model					
	Climate		Climate + Soil		Climate + Soil + Human	
	Percent Contribution	Permutation Importance	Percent Contribution	Permutation Importance	Percent Contribution	Permutation Importance
**Bio4**	**49.6**	**37.2**	**43.1**	**42**	**36.5**	**47.7**
**PET**	**15**	**4.6**	**10.7**	0.4	**15.2**	0.3
**Solar_rad**	**14.7**	**17.8**	3.8	2.2	2.4	2.7
**AI**	**13**	**19.5**	0.3	1.3	0.9	1
Wind_spd	4.2	1.7	0.6	0.7	2	1
Bio8	0.9	3.7	0.6	5.3	2.2	5.4
**Bio9**	1.5	**14.2**	1.8	**21.1**	1.4	**15.9**
Bio15	0.3	0.7	0	0	1.4	0
Bio16	0.8	0.5	0.3	0.4	0.5	1
**PHIHOX**	-	-	**25.6**	**17.5**	**23.4**	**18.9**
CEC	-	-	0.8	0.9	3.1	1.4
**CLYPPT**	-	-	**9.3**	**5.9**	**7.7**	3.5
ORCDRC	-	-	0.6	0.3	0.6	0.5
CRFVOL	-	-	0.3	0.3	0.4	0.3
AWCh	-	-	2.2	1.5	2.3	0.3
HII	-	-	-	-	0.1	0.1
AUC	0.947		0.958		0.958	
Sensitivity	1		1		0.998	
TSS	0.589		0.559		0.585	

* Bio4: temperature seasonality (standard deviation * 100); PET: potential evapotranspiration (mm); Solar_rad: solar radiation (kJ m^−2^ day^−1^); AI: aridity index; Wind_spd: wind speed (m s^−1^); Bio8: mean temperatures of the wettest quarter (°C); Bio9: Mean temperature of the driest quarter (°C); Bio15: precipitation seasonality (C of V); Bio16: precipitation of wettest quarter (mm), PHIHOX: soil pH × 10 in H_2_O; CEC: cation exchange capacity in cmolc/kg; CLYPPT: soil texture fraction clay in percent; ORCDRC: soil organic carbon content in g per kg; CRFVOL: coarse fragments volumetric in percent; AWCh: Available soil water capacity; HII: human influence index.

## Data Availability

Data are contained within the article or Supplementary Material.

## Supplementary Materials

The following are available online at https://www.mdpi.com/2079-7737/10/3/203/s1, Table S1: Occurrence data, Table S2: Environmental Data, Table S3: Climate correlations, Table S4: Soil correlations, Table S5: Biome data; Figure S1: Jack-knife test for evaluating the relative importance of predictor variables for *Prosopis juliflora* globally (generated by MaxEnt climate + soil model). Figure S2: Response curves of the less important climatic and edaphic predictor variables of *Prosopis juliflora* generated by MaxEnt (Climate + Soil model).
