# Supplementary material for: Global Invasion Risk Assessment of Prosopis juliflora at Biome Level: Does Soil Matter?
Source: Biology (Basel). 2021 Mar 9;10(3):203. doi: 10.3390/biology10030203 (PMC7999975; doi:10.3390/biology10030203)
Supplement: Supplementary file 1 [file biology-10-00203-s001.zip › Supplementary B.docx]

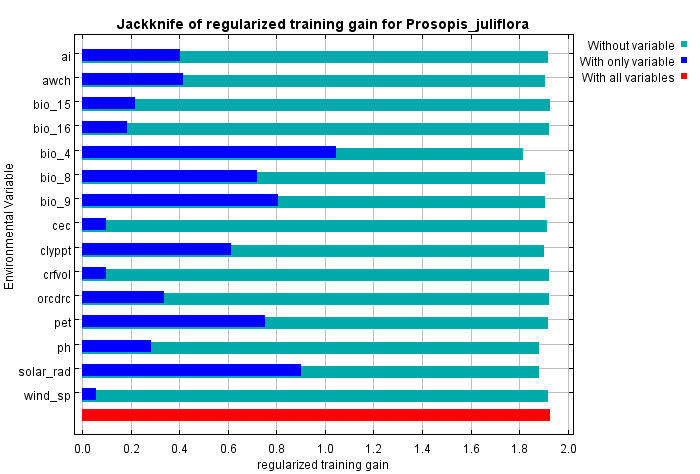


Fig. S1. Jack-knife test for evaluating the relative importance of predictor variables for *Prosopis juliflora* globally (generated by MaxEnt climate + soil model).


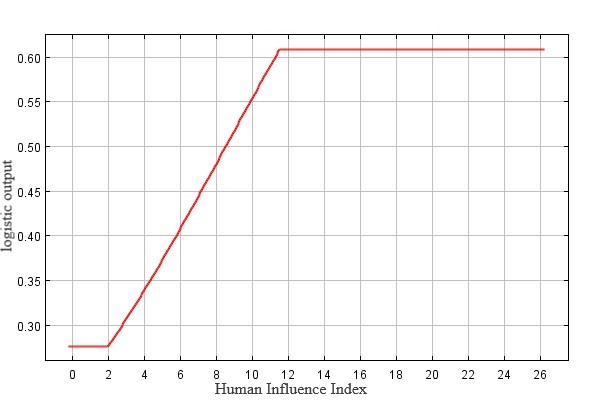

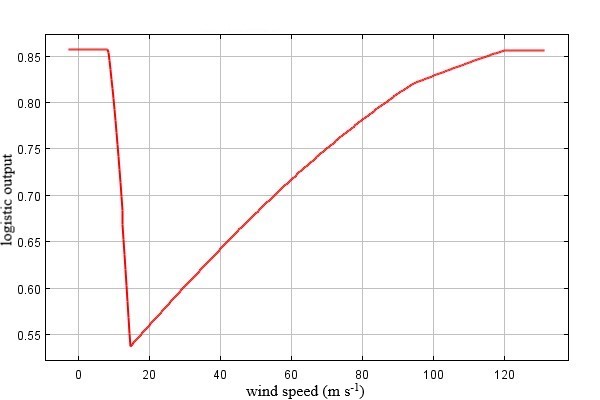

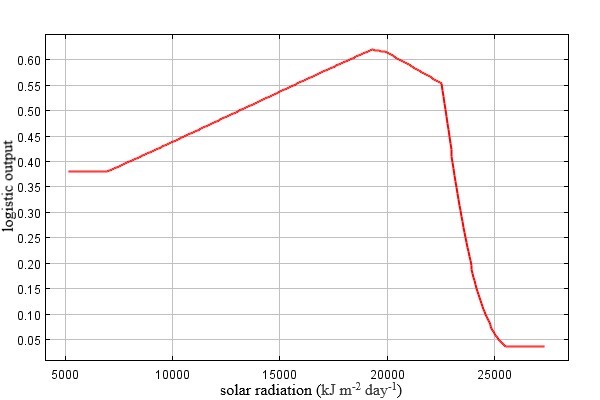

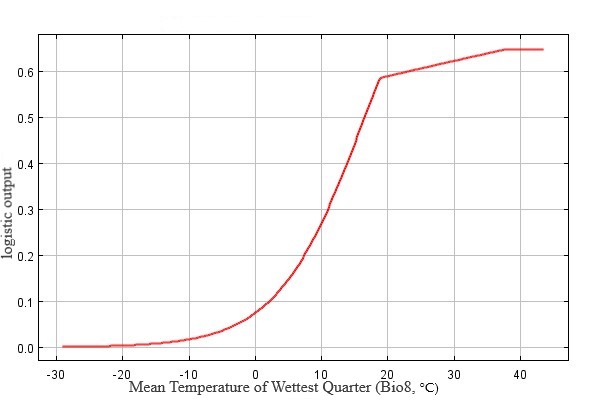

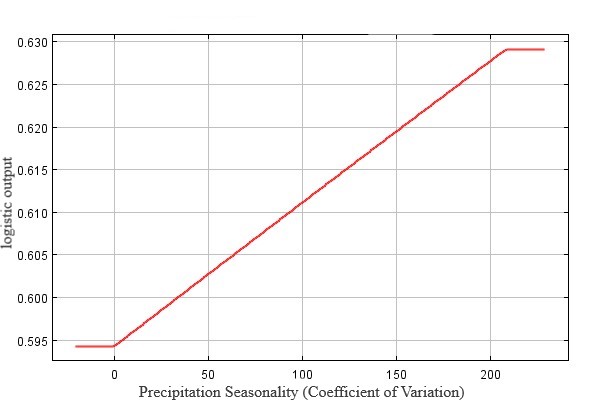

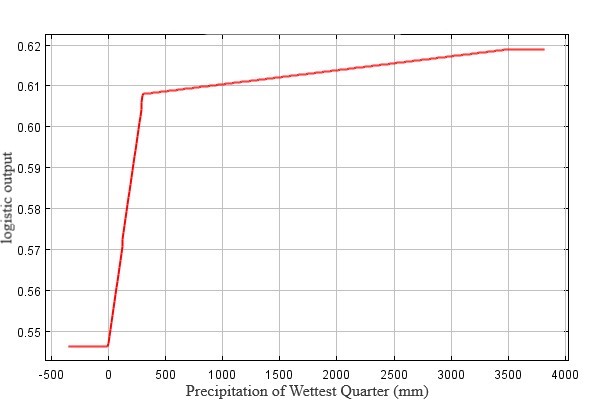

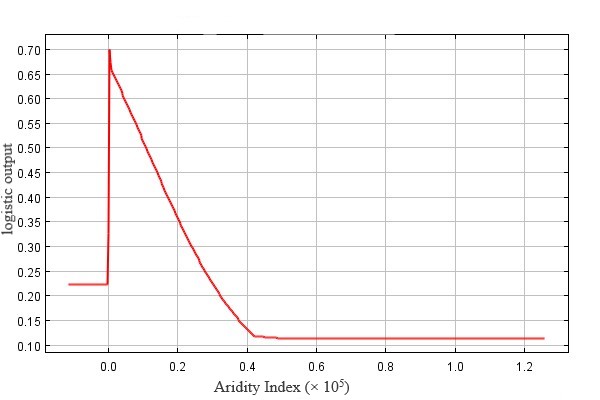

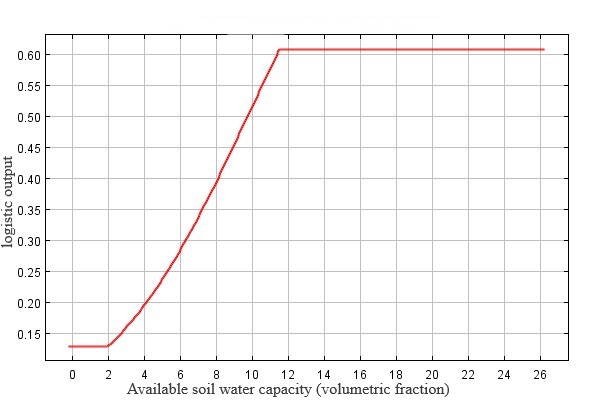

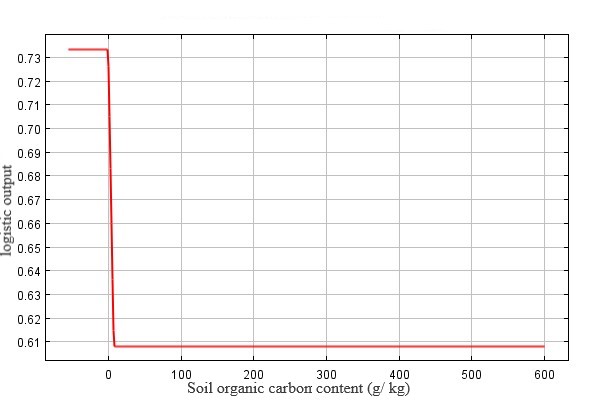

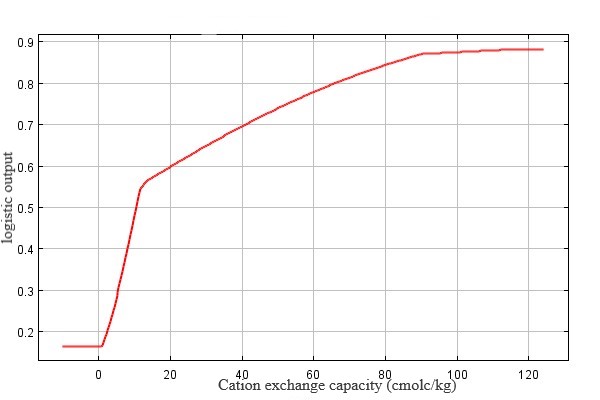

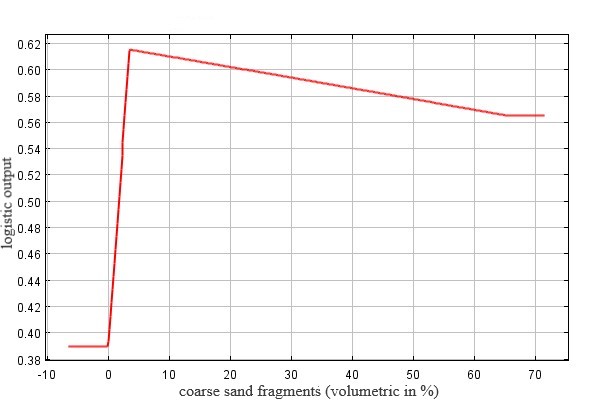


Figure S2. Response curves of the less important climatic and edaphic predictor variables of *Prosopis juliflora* generated by MaxEnt (Climate + Soil model).
